# Gene Expression, Non-Coding RNA, and Circular RNA Alterations in Patients with T-Prolymphocytic Leukemia

**DOI:** 10.3390/cancers18152442

**Published:** 2026-07-29

**Authors:** Vanessa Rebecca Gasparini, Silvia Orsi, Alessia Buratin, Elisa Rampazzo, Giulia Calabretto, Elena Buson, Alberto Caregari, Cristina Vicenzetto, Gregorio Barilà, Eleonora Roncaglia, Roberto Merlo, Livio Trentin, Monica Facco, Laura Pavan, Gianpietro Semenzato, Enrico Gaffo, Antonella Teramo, Renato Zambello, Stefania Bortoluzzi

**Affiliations:** 1 Hematology Section, Department of Medicine, University of Padova, 35128 Padova, Italy; 2 Veneto Institute of Molecular Medicine (VIMM), 35129 Padova, Italy; 3 Department of Biology, University of Padova, 35131 Padova, Italy; 4 Department of Molecular Medicine, University of Padova, 35131 Padova, Italy; 5 Cardiology Section, Department of Cardiac Thoracic Vascular Sciences and Public Health, University of Padova, 35129 Padova, Italy; 6 Hematology Unit, San Bortolo Hospital, 36100 Vicenza, Italy; 7 Department of Surgery, Oncology and Gastroenterology, University of Padova, 35131 Padova, Italy

**Keywords:** T-PLL, gene expression, non-coding RNA, circular RNA

## Abstract

**Background/Objectives**: Identifying molecular liabilities and understanding disease heterogeneity are prerequisites for advancing therapies in T-cell prolymphocytic leukemia (T-PLL), a rare T-cell malignancy with a poor prognosis. **Methods**: RNA-seq profiling of T-PLL samples (*n* = 10) and the normal counterpart (*n* = 5) allowed us to report gene expression and pathway alterations in malignant cells, revealing that non-coding, antisense and circular RNA expression is profoundly altered in T-PLL. **Results**: T-PLL displayed activation of several oncogenic pathways, particularly PI3K/AKT/mTOR and Wnt, suppression of healthy T-cell activities and cell death escape. Tumor suppressor lncRNAs (*NEAT1*, *MIAT* and *LUCAT1*) with reduced expression and upregulated oncogenic pro-proliferative lncRNAs (*FIRRE*, *TERC*, *XIST* and *PVT1*) were identified. CircRNAs ectopically expressed in T-PLL included circSEMA4B and circSATB1, linked to the Wnt pathway, circFIRRE and oncogenic circPVT1 and circFKBP5. Focusing on five genes with validated recurrent oncogenic variants (*STAT5B*, *JAK3*, *ATM*, *KMT2C*, and *ARID1A*), we investigated genotype/phenotype relations. A multiple predictor linear model suggested potential links between driver variants and alterations in gene and circRNA expression, including association between *STAT5B* mutations and *LTF* upregulation, *JAK3* lesions and increased *PLXNA4* expression along with *CCR4* suppression. **Conclusions**: Our transcriptomic profiling and genotype–phenotype association analysis identified specific genes, non-coding RNAs, pathways and candidate genotype-associated transcriptional signatures that warrant further investigation as potential targets for the development of new therapeutic approaches for this rare and heterogeneous malignancy.

## Simple Summary

The high heterogeneity of the T-PLL molecular background considerably challenges the identification of treatments for this incurable disease. The emerging roles of lncRNAs and circRNAs in hematological malignancies prompted their study in the context of T-PLL. Here, we identified gene, lncRNA, and circRNA expression alterations and aberrant pathway activations in T-PLL, combining genomic and transcriptomic data to explore the links between driver variants and expression alterations. This study opens new lines of investigation into common pathways (e.g., the Wnt pathway) hit in T-PLL patients that could be pharmacologically targetable to potentially expand therapeutic options in the future.

## 1. Introduction

T-cell prolymphocytic leukemia (T-PLL) is an extremely aggressive malignancy of mature T lymphocytes characterized by a rapid clinical course and resistance to conventional chemotherapy [1,2,3]. Humanized CD52-antibody alemtuzumab is effective as first-line therapy [3,4,5], but relapses are inevitable without consolidation with allogeneic transplantation, which is feasible only in a restricted subset of patients [4].

Leukemic T-PLL cells are characterized by a complex karyotype, with alterations of chromosome 14 leading to the overexpression of *TCL1A* or *MTCP1* proto-oncogenes [5,6,7] and deletions of 11q with *ATM* loss [7]. Other recurrent alterations involve chromosome 8, including *MYC* gain [5]. Genomic studies have shown that almost 60% of the lesions affect the JAK/STAT axis, with *JAK3* (30–42%), *STAT5B* (19–36%) and *JAK1* (6–8%) being the most recurrently mutated genes [8,9,10]. In addition, mutations in *ATM*, *TP53*, DNA repair/checkpoint proteins and epigenetic regulators are recurrent [7,9].

Conversely, the transcriptomic profile of T-PLL patients has been rarely studied. Array-based gene expression profiling in 5 T-PLL patients identified putative genes involved in lymphomagenesis, cell cycle regulation, apoptosis and DNA repair [11]. A larger study also included genes associated with proliferation, immune responses, and signal transduction intermediates [7]. Defined microRNA expression patterns were associated with worse prognosis [12,13,14]. In the last few years, new classes of non-coding RNA such as long non-coding RNA (lncRNA) and circular RNA (circRNA) have been extensively studied in cancer and in hematological conditions for their involvement in pathogenesis, progression and therapeutic resistance [15,16]. However, scattered data of these molecules in the context of T-PLL are available.

Here, we provide new data on the molecular makeup of T-PLL, leveraging multi-level omics data, which encompass comprehensive transcriptomic analysis integrated with the definition of patient genetic aberrations. In this way, we extend the knowledge about the most dysregulated genes and pathways, disclose alterations of non-coding RNAs and circRNAs in malignant T-PLL cells, and model the link between recurrent driver mutations with gene and circRNA expression patterns in this rare and challenging disease.

## 2. Materials and Methods

### 2.1. Cohort of Study

The study comprises 19 CD4+ T-PLL patients and 9 healthy controls (CTR). All the patients were recruited at the Hematology Unit of Padua University Hospital and met the diagnostic criteria for T-PLL (Supplementary Table S1) [17]. The study was performed according to the Helsinki Declaration and approved by the Padua Provincial Ethical Committee for Clinical Trials (CESC code 5229/AO/21; URC code AOP2436). All the patients gave written informed consent before inclusion in the study. Healthy controls were recruited from the Department of Transfusion Medicine of Padua University Hospital. Details about sample collection and purification, as well as about mutation screening are described in the Supplementary Methods S1.

### 2.2. RNA-Seq Profiling

RNA was extracted from peripheral blood mononuclear cells (PBMC) of CD4+ T-PLL patients and CD4+ cells of CTR using the RNeasy kit (Qiagen, Hilden, Germany) following the manufacturer’s instructions. RNA sequencing (RNA-seq) was performed using the Illumina platform (paired-end, 60 million reads on average per sample, Ribozero Gold rRNA depletion kit; Illumina, San Diego, CA, USA). RNA-sequencing data are available at Gene Expression Omnibus (GSE281431).

### 2.3. Gene and circRNA Expression Estimation from RNA-Seq

Expressed genes and circRNAs were detected with CirComPara2 v0.6.3 [18], implementing data quality control and filtering, read alignment to the reference genome and linear and circular transcriptome reconstruction, respectively, using Hisat2 and the combination of seven backsplicing detection methods. More details about the filtering, differential expression and functional enrichment analyses are described in the Supplementary Methods S2.

### 2.4. RT-qPCR for Expression Quantification

Quantitative real-time PCR (RT-qPCR) has been performed to confirm dysregulated expression of transcripts in T-PLL. Primers for circRNAs were designed with Primer Blast (Supplementary Table S2; Sigma-Aldrich, Saint Louis, MO, USA) to amplify the backsplice junction region. PCR product starting from cDNA pre-treated with RNase R (ThermoFisher Scientific, Waltham, MA, USA), to optimize circRNA detection, was analyzed with Sanger sequencing to confirm the amplification of the correct region. Gene expression quantification was obtained on QuantStudio 5 Real-Time PCR System (ThermoFisher Scientific, Waltham, MA, USA) and analyzed using the comparative threshold cycle method (ddCt) with *GAPDH* as the reference gene.

### 2.5. Variant Calling and Validation

Expressed candidate driver variants were inferred from RNA-seq data; variant annotation and selection is detailed in Supplementary Methods S3. Driver variant prediction was obtained by Cancer Genome Interpreter, and the functional enrichment of mutated driver genes was calculated (Supplementary Methods S4).

Selected candidate variants were prioritized for validation according to their gene function and variant allele frequency in order to confirm their presence in the entire cohort. Validation was performed with Sanger sequencing using custom primers designed with Primer Blast (Supplementary Table S2; Sigma-Aldrich, Saint Louis, MO, USA). Purified PCR products were sequenced on an ABI 3130 sequencer (Applied Biosystems, Waltham, MA, USA), and the results were analyzed with NCBI BLASTN (v.2.15.0+) and ChromasPro (v.2.1.20.1).

### 2.6. Modeling Gene and circRNA Expression Relation with Genetic Lesions

To identify significant relations between T-PLL cells’ genotype, considering driver genes hit by point mutations, and phenotype, in terms of gene and circRNA expression profile, a linear expression model fitted with Limma has been used (Supplementary Methods S5).

## 3. Results

### 3.1. Clinical and Molecular Features of the T-PLL Cohort and Study Design

PBMC from 10 CD4+ T-PLL patient samples (Table 1) and sorted CD4+ cells from age-matched healthy donor samples (*n* = 5), as normal counterparts (CTR), were profiled by RNA-seq.

Standard screening by Sanger sequencing detected *STAT5B* and *JAK3* variants in six and two cases, respectively. The median age at diagnosis was 69 years. The leukemic clone phenotype resulted in CD4+ and CD7+, with a variable expression of CD25 and CD26 markers. All cases presented a clonal expansion according to the molecular evaluation of the TCR Gamma gene rearrangement, while Vbeta 7.1 expansion was recurrent in 25% of cases. All patients presented with a high white blood cell count, marked lymphocytosis, reduced hemoglobin levels, lymphadenopathy and hepatosplenomegaly (Supplementary Table S1).

### 3.2. Aberrant Gene Expression and Pathway Activity in T-PLL

CirComPara2 analysis extracted expression profiles of both genes (14,912) and circular RNAs (6469) in T-PLL and the normal counterpart from RNA-seq data.

**Figure 1:**
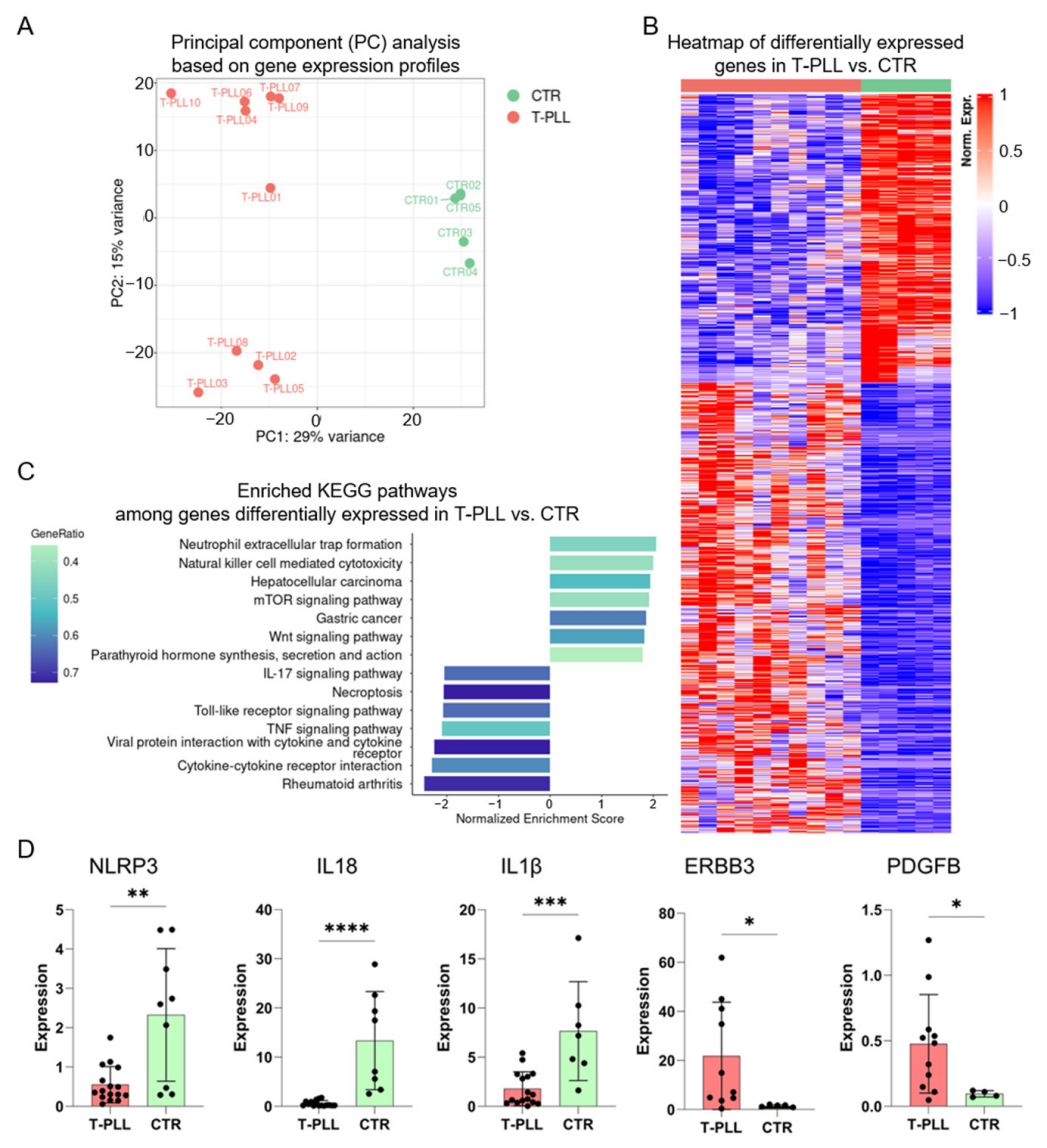
Aberrant gene expression and pathway activation in T-PLL. (**A**) Principal component analysis (PCA) of 10 T-PLL and 5 control (CTR) samples according to gene expression profiles; (**B**) heatmap of expression profiles (average clustering based on Spearman correlation-based distance; scaled expression) and (**C**) enriched KEGG pathways according to GSEA of 3693 differentially expressed genes comparing T-PLL with CTR (adj. *p*-value < 0.01); (**D**) RT-qPCR quantification of gene expression in an extended cohort of 16 T-PLL cases and 9 CTR (*, **, ***, **** Mann–Whitney *p*-value < 0.05, 0.01, 0.001, 0.0001).

According to principal component analysis (PCA) based on gene expression profiles (Figure 1A), T-PLL samples were separated from CTR samples. A direct comparison identified 2009 genes differentially expressed, with 60% being abnormally upregulated in malignant cells (Figure 1B and Supplementary Table S3). Differential expression analysis showed an increased expression of several cancer-associated genes in the T-PLL samples, including *ERBB3*, *PDGFB* and *MYC* (Supplementary Table S3). Gene Set Enrichment Analysis (GSEA; Figure 1C) identified enrichment of several cancer-related pathways, particularly PI3K/AKT/mTOR and Wnt signaling. In line with the enrichment of Wnt signaling, *LRP5* and *FZD8* encode a Wnt co-receptor and receptor, respectively, whereas *DAAM1* acts downstream of non-canonical Wnt signaling and regulates actin cytoskeleton remodeling. Additionally, a series of pathways and functions active in healthy T-cells were suppressed in T-PLL such as antigen processing and presentation, apoptosis, cytokine regulation and T cell differentiation (Supplementary Figure S1 and Figure 1C). *ERBB3* and *PDGFB* were selected for RT-qPCR validation based on their expression levels, degree of dysregulation, and potential biological relevance, and their upregulation was confirmed in the extended cohort (Figure 1D). RT-qPCR also confirmed the downregulation of *IL1β* and *NLRP3*. Although the decrease in *IL18* did not reach statistical significance in the RNA-seq cohort, its significant downregulation was confirmed by RT-qPCR in the extended cohort, consistent with the direction of change observed in the sequencing data (Figure 1D).

In conclusion, the aberrant expression of coding transcripts in T-PLL indicated the activation of distinct oncogenic signaling pathways, particularly PI3K/AKT/mTOR and Wnt, in addition to the suppression of healthy T-cell activities and the escape of multiple cell death mechanisms.

### 3.3. Refining the T-PLL Transcriptomics: Aberrant Non-Coding and Circular RNA Expression

To give new insight about the transcriptomic profile of T-PLL patients, we investigated the expression of antisense long non-coding RNAs and circular RNAs in T-PLL.

**Figure 2:**
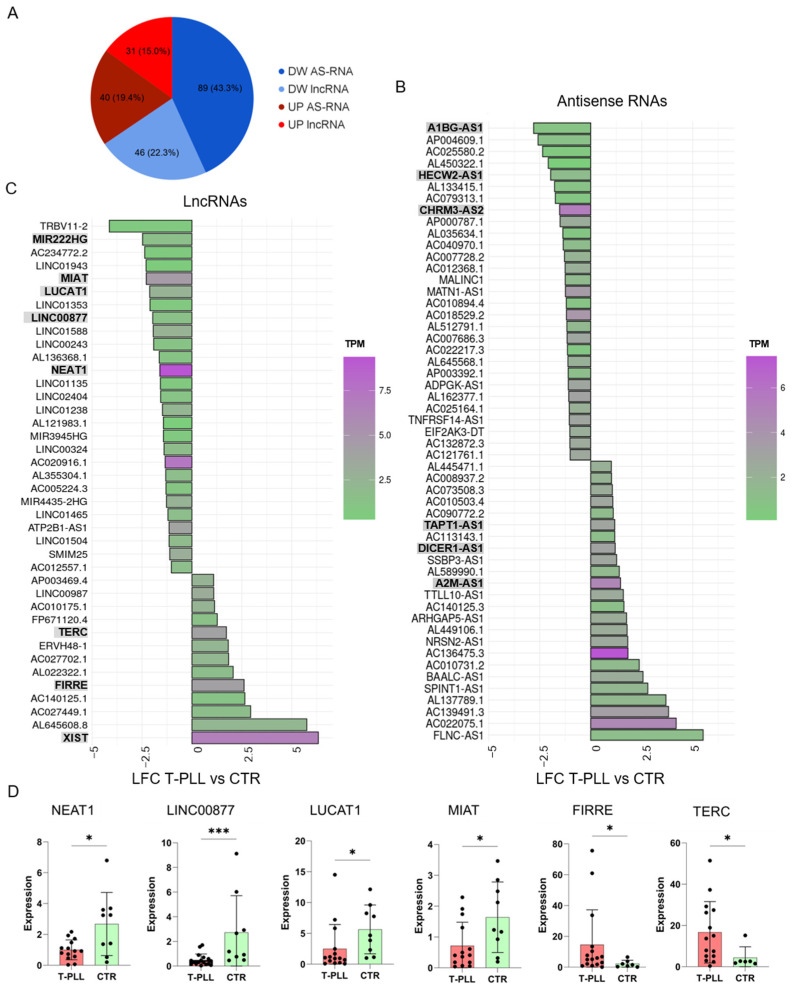
Long non-coding RNAs (lncRNAs) and antisense RNAs with altered expression in T-PLL. (**A**) Pie chart of number and percentage of up- and downregulated RNAs; waterfall plots of (**B**) antisense RNAs and (**C**) lncRNAs with significant differential expression between T-PLL and CTR (adj. *p*-value < 0.01; only transcripts with |LFC| > 1 are shown; the bar color indicates the average expression as TPM (transcripts per million reads); bold text with light grey background indicates the RNAs cited in the main text; (**D**) RT-qPCR quantification of transcript expression in an extended cohort of 16 T-PLL cases and 9 CTR (*, ***, Mann–Whitney *p*-value < 0.05, 0.001).

We found 129 antisense (AS) and 77 long non-coding RNAs (lncRNAs) with altered expression, prevalently (69% and 60%) downregulated, in T-PLL (Figure 2A). Among the differentially expressed antisense RNAs (Figure 2B), *A1BG-AS1*, a tumor suppressor in solid cancer [19], was among the most aberrantly downregulated in T-PLL. Conversely, *A2M-AS1* and *DICER1-AS1*, with oncogenic roles in other settings [20,21], and the less characterized *TAPT1-AS1* were overexpressed in T-PLL.

Several lncRNAs were dysregulated (Figure 2C), including *MIR222HG*, *MIAT*, *LUCAT1* and *LINC00877* within the most downregulated. RNA-seq and RT-qPCR (Figure 2C,D) concordantly showed the downregulation of *NEAT1*, a nuclear paraspeckle lncRNA that acts as a tumor suppressor in leukemia [22,23]. RT-qPCR (Figure 2D) also confirmed the downregulation of *MIAT*, *LUCAT1* and *LINC00877*. These lncRNAs were firstly associated with the initiation of metastasis and proliferation in several malignant tumors [24], while *LUCAT1* downregulation in acute lymphoblastic leukemia [25] and of *LINC00877* in mantle cell lymphoma [26] were recently described.

Among the most upregulated lncRNAs (Figure 2C), we found *FIRRE* and *TERC* (Figure 2D), previously linked to pro-proliferative functions [27], *XIST*, an important regulator of cell growth and development, and *PVT1*, a well-known lncRNA under control of p53, that promotes tumorigenesis, boosting MYC expression [28], which is increased in our data (Supplementary Table S3).

**Figure 3:**
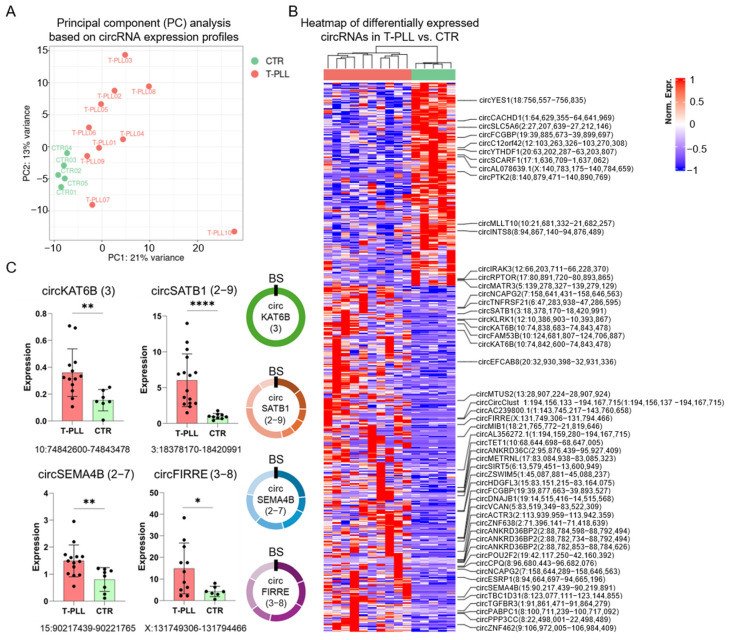
The aberrant circRNAome of T-PLL. (**A**) Principal component analysis (PCA) of 10 T-PLL and 5 control (CTR) samples according to circRNA expression profiles; (**B**) heatmap of 261 circRNAs differentially expressed comparing T-PLL with CTR (adj. *p*-value < 0.01; names are shown for circRNAs with |LFC| ≥ 2 and high expression with backsplice junction counts >150; average clustering based on Euclidean distance, scaled expression); (**C**) structure (exons and backsplice junction, BS) and RT-qPCR quantification of circRNA expression in an extended cohort of 16 T-PLL cases and 9 CTR (*, **, **** Mann–Whitney *p*-value < 0.05, 0.01, 0.0001).

The examination of circRNA expression in T-PLL provided for the first time a comprehensive profile of the circRNAome aberrancies in this malignancy. Descriptive analysis showed that T-PLL samples can be discriminated from CTR exclusively using circRNA expression profiles (Figure 3A). In line, 261 circRNAs were significantly differentially expressed, prevalently (71%) upregulated, in T-PLL compared to CTR (Figure 3B and Supplementary Table S4).

Among the upregulated circRNAs, 85 were ectopically expressed in T-PLL, including circKAT6B, circPVT1 and circFKBP5 with known oncogenic functions [29,30,31]. We selected four significantly upregulated circRNAs, namely circKAT6B, circSATB1, circSEMA4B, and circFIRRE, based on robust back-splice junction read support, relevant fold change, and prior cancer- or T-cell-related annotations and validated their differential expression compared to CTR (Figure 3C). The abnormal overexpression of both the linear lncRNA *FIRRE* (Figure 2D) and circFIRRE has been confirmed, and the circular to linear proportion (CLP [32]) rose from 0.38 in CTR to 0.55 in T-PLL.

Most of the downregulated circRNAs (Figure 3B), comprising circMETRNL, circIRAK3, circPOU2F2, circACTR3, circZNF638 and circVCAN, were poorly characterized.

### 3.4. Association of Driver Variants with Distinct Gene and circRNA Expression Profiles

The next aim of the study was to obtain a better characterization of the disease heterogeneity, by defining the association of driver mutations with the transcriptome profile in T-PLL. The genomic state of patients was inferred from RNA-seq data by identification of expressed driver variant alleles with putative pathogenic relevance.

**Figure 4:**
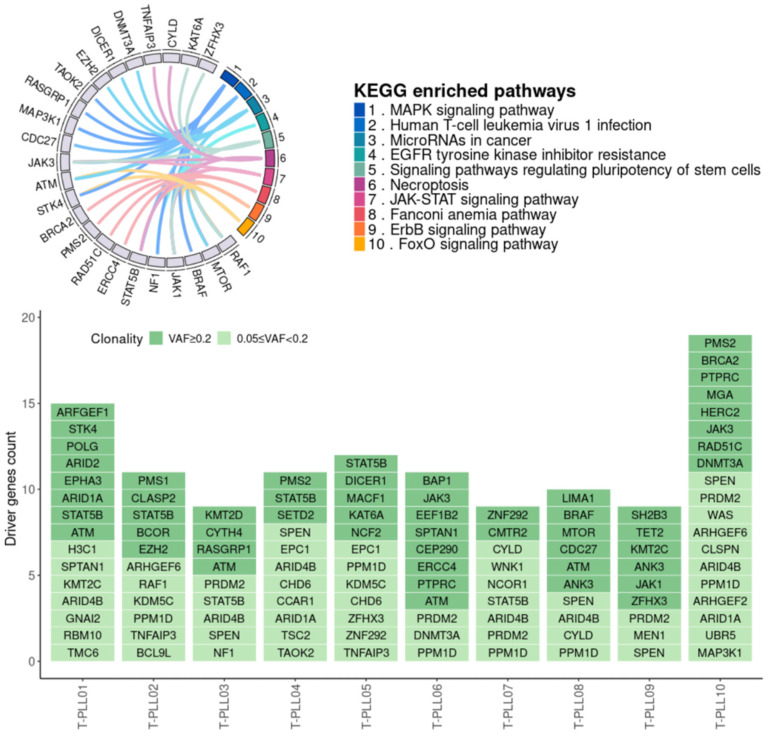
Genes and pathways hit by expressed candidate driver variants inferred from RNA-seq data in T-PLL. Circos plot indicates the links between mutated genes and significantly enriched KEGG pathways; the barplot below shows the genes with driver variants detected in each patient, indicating the estimated variant allele frequency (VAF) class for each mutation.

We detected 1991 rare variants per patient, on average, with a VAF at least 5% in leukemic cells. We found 126 driver variants in 73 genes according to CGI analysis (Supplementary Tables S1 and S5). The validation by Sanger sequencing of 18 selected variants corroborated the robustness of these data (Supplementary Table S6 and Supplementary Figure S2). Patients presented a complex molecular profile harboring from 12 to 26 driver variants, with multiple oncogenic variants with an estimated VAF of at least 0.2 in most cases (Figure 4). Mutations were found in genes previously associated with T-PLL (*ATM*, *JAK3*, *KMT2C*, *PTPRC*) and in others not previously described in this malignancy (*ANK3*, *ARID1A*, *CMTR2*), which were validated. *STAT5B* was mutated in six cases, with two also carrying *ATM* variants. In one case, a low VAF (0.07) *STAT5B* variant coexisted with variants of *KMT2D*, *RASGRP1*, *CYTH4*, and *NF1* genes, which were not previously described in T-PLL. *ATM* inactivating variants were detected in four cases. *JAK* genes were mutated in three cases (all *STAT5B* negative), with *JAK3* and *JAK1* variants coexisting in two. Ten KEGG pathways were enriched in driver-mutated genes (Figure 4), with MAPK signaling, chemokine signaling, EGFR tyrosine kinase inhibitor resistance and the JAK-STAT signaling pathway representing the hallmarks of the disease.

**Figure 5:**
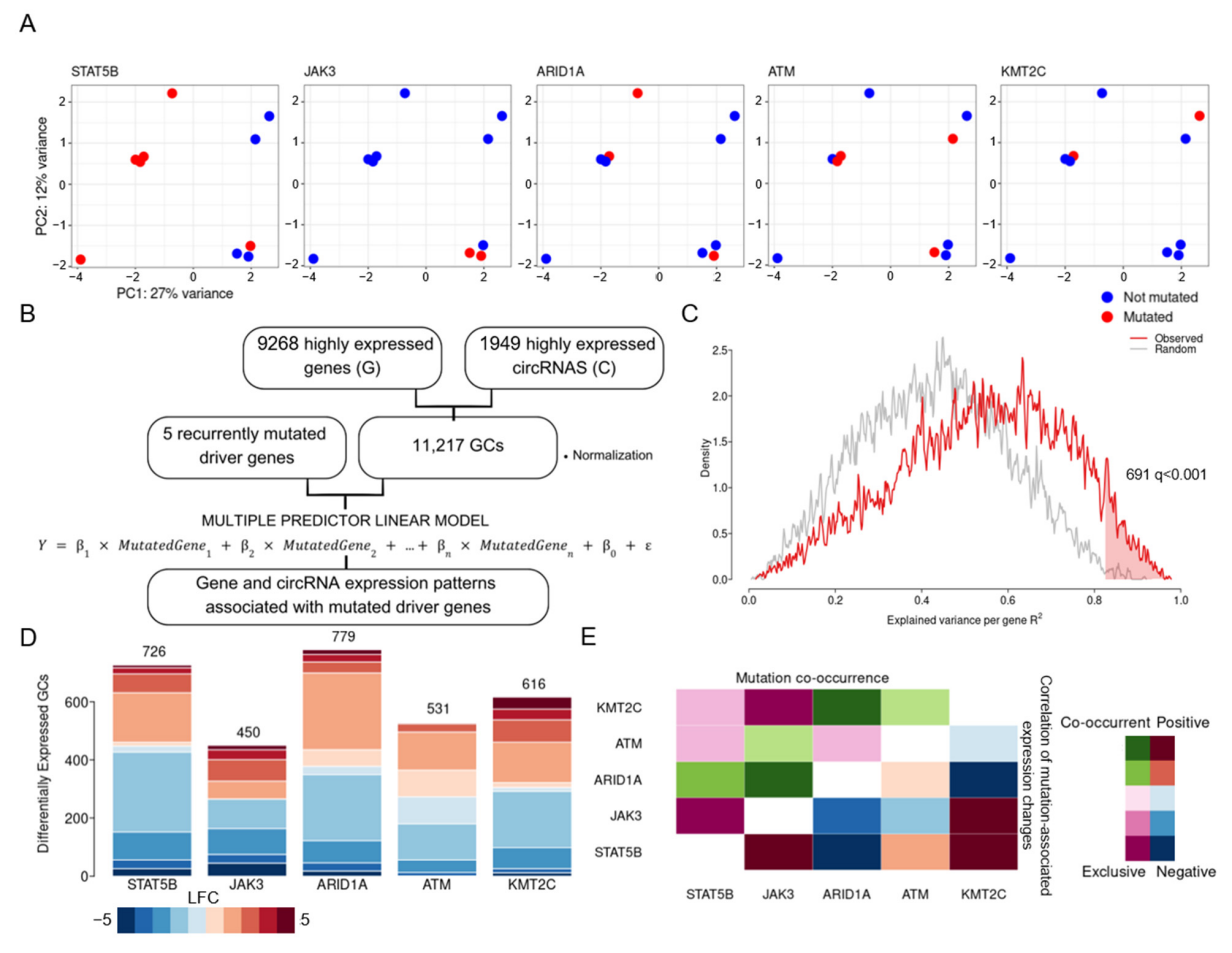
A model of the association between driver gene mutations and gene and circRNA expression profile alteration in T-PLL. (**A**) Principal component analysis (PCA) of 10 T-PLL cases according to gene and circRNA expression profiles; for each of the mutated genes considered (*STAT5B*, *JAK3*, *ARID1A*, *ATM* and *KMT2C*), a plot shows as red and blue dots the samples with and without the gene mutation, respectively; (**B**) overview of the strategy used to identify genes and circRNAs whose expression was most strongly associated with the presence of the investigated mutations (see the main text and Supplementary Materials for more details); (**C**) per-feature variance explained (R^2^) by the five genomic alterations (red) and empirical null obtained by permuting genotype labels and refitting the same model (grey). Shaded: 691 features above the null-derived threshold (R^2^ > 0.82, q < 0.001); (**D**) number of genes and circRNAs differentially expressed in the presence of each mutation, indicating separately the numbers of up-and downregulated elements with different LFC classes; (**E**) pairwise relationships among the five recurrently mutated genes. Lower triangle: Pearson correlation between mutation-associated expression-change profiles (per-gene coefficient vectors from the multiple-predictor linear model across 1843 features); red, concordant; blue, opposing. Upper triangle: mutation co-occurrence across the cohort (odds ratios from the binary mutation–status matrix); green, co-occurrent; pink, mutually exclusive. Because co-occurring lesions are collinear predictors, the two triangles are not independent.

Considering the heterogeneous data obtained, we performed an exploratory analysis of how recurrent orthogonally validated variants in *STAT5B*, *ATM*, *ARID1A*, *KMT2C* and *JAK3* relate to gene and circRNA expression. Expression profiles of 9268 genes and 1949 circRNAs robustly expressed were normalized and merged in an integrated expression matrix. The PCA of patient sample separation indicated that cases sharing the same lesion could have a similar expression profile (Figure 5A). Because several of these lesions co-occur within individual cases, we did not attempt to isolate single-gene effects univariately; instead, a multiple-predictor linear model including all five genotypes was fitted per feature, so that associations are estimated while adjusting for co-occurring variants (Figure 5B). To distinguish genuine associations from those expected under the limited sample size and large feature space, per-feature R^2^ was benchmarked against an empirical null generated by permuting genotype labels and refitting the identical model; here, 691 features exceeded the null-derived threshold (R^2^ > 0.82, q < 0.001), indicating collective genotype-associated structure above chance rather than variance attributable to individual lesions (Figure 5C). Thus, 1843 genes or circRNAs in the model were significantly differentially expressed in mutated samples (Figure 5D and Supplementary Table S7). Considering mutation co-occurrence (Figure 5E), the couples *JAK3/STAT5B* and *JAK3/KMT2C*, both mutually exclusive in our patient set, were linked to similar expression profiles. Conversely, *ARID1A* lesions were associated with a distinct profile, although this lesion co-occurred with both *KMT2C* and *JAK3* and partially with *STAT5B*.

**Figure 6:**
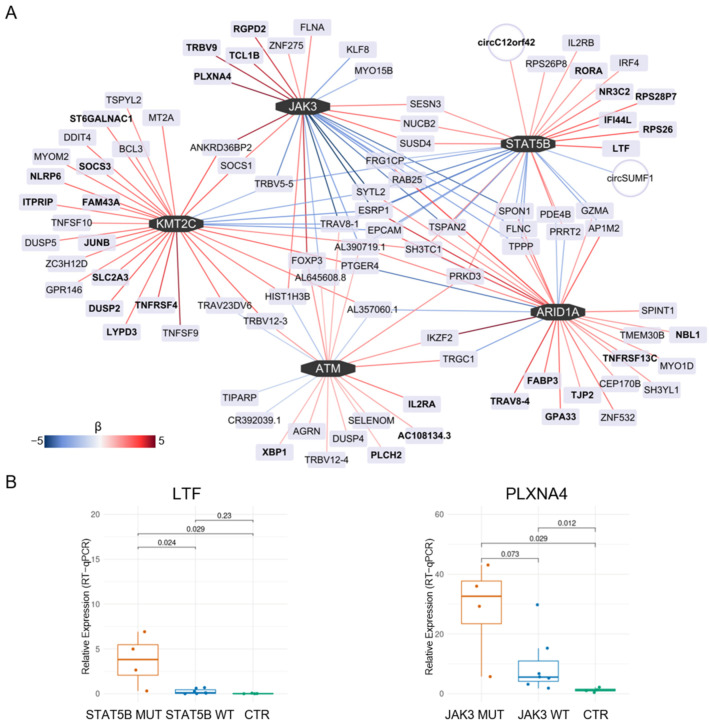
Gene and circRNA expression profile associated with distinct driver gene variants in T-PLL. (**A**) Network representation of the strongest associations detected between driver variants and gene and circRNA expression changes. For each mutated gene, the 25 genes or circRNAs whose expression was most strongly associated with mutation status according to the linear model are shown; edge colors indicate linear model β coefficients; genes and circRNAs highlighted in bold are significantly associated with only one mutated gene, according to the model. (**B**) Relative expression quantified by RT-qPCR (DDCt method, normalized to GAPDH) in T-PLL cases with and without the driver variant (4 *STAT5B* mutated and 7 with wild-type STAT5B for *LTF* and 4 *JAK3* mutated and 7 with wild-type *JAK3* for *PLXNA4*) and 4 CTR (The Wilcoxon test *p*-value is reported for each comparison).

The network in Supplementary Figure S3 shows, for each lesion, the 50 genes or circRNAs whose expression was most strongly associated with mutation status according to the linear model. Of the 51 genes associated with 2–4 mutated genes, 25 showed consistent and 26 showed opposite behavior. For instance, *ESRP1* was upregulated in cases with *ARID1A* mutation and downregulated in *STAT5B-* and *JAK3*-mutated cases, whereas *FOXP3* was upregulated in *JAK3-* and *ATM*-mutated cases. *STAT5B-* and *JAK3*-mutated cases shared the upregulation of *NUCB2*, *SESN3* and *SUSD4*. All the circRNAs and most genes (126, 72%) were instead linked with only one lesion. A reduced network showing, for each lesion, the 25 genes or circRNAs whose expression was most strongly associated, either positively or negatively, with the corresponding mutation status according to the multiple-predictor linear model, is presented in Figure 6A. *ATM* mutation was linked to the upregulation of *IL2RA*. *ARID1A* and, at a lesser extent, *ATM* lesions were linked to an upregulation of *IKZF2. TNFSF9* and *TNFRSF4* upregulation were present in cases with *KMT2C* mutation. Of particular interest, *STAT5B* lesions were associated with the upregulation of *LTF*, while in *JAK3*-mutated cases, *PLXNA4* was upregulated. These latter specific upregulations were confirmed in the extended cohort, further strengthening our findings (Figure 6B).

## 4. Discussion

In this study, we report gene expression and pathway dysregulation, along with aberrations in non-coding and circular transcriptome in T-PLL. By integrating multi-modal data from RNA-seq, we gained a deeper understanding of the heterogeneity in expression profiles associated with this malignancy, providing new insights into links between driver lesions and transcriptome alterations.

We confirmed the overexpression of *TCL1A* and *MYC* and uncovered the alteration of the Wnt signaling pathway. We also observed the *BCL2* dependency of T-PLL through the overexpression of *BCL2L1*, pharmacologically inhibited by Venetoclax [33]. This drug has been tested in a recent clinical trial with variable success rates, thus requiring a prior patient stratification for a better benefit from the treatment [34,35,36].

Additionally, we observed the downregulation of several genes in the IL-17 signaling pathway in T-PLL. The confirmed low expression of *IL1β*, *IL18* and *NLRP3* in leukemic cells indicates their escape from Fas-induced apoptosis [37] and pyroptosis [38], thus opening new lines of investigation about the pharmacological restoration of the *NLRP3/IL1β* axis [39].

Beyond gene expression dysregulation, we identified non-coding RNA and circRNA with altered expression in T-PLL. Most of the non-coding RNAs were downregulated, including *NEAT1*, *MIAT*, *LUCAT1* and *LINC00877,* whose significantly reduced expression in T-PLL was validated. In acute leukemia [40], it has been demonstrated that the nuclear paraspeckle lncRNA *NEAT1* translocates to the cytoplasm suppressing Wnt signaling; thus, its downregulation observed in our cohort might be potentially relevant also in the context of T-PLL as a contributing factor to Wnt signaling hyperactivation. In addition, the circRNAome of T-PLL was found to be highly dysregulated with the ectopic expression in T-PLL of several circRNAs with oncogenic functions [29,30,31]. Amongst the circRNAs with validated upregulation in T-PLL, we detected circSEMA4B, which was previously shown to inhibit *IL1β* expression acting through the Wnt signaling pathway [41], suggesting an axis worth further investigation in T-PLL. We also confirmed the upregulation of circSATB1 in T-PLL, as observed in T-cell acute lymphoblastic leukemia (T-ALL). This circRNA derives from *SATB1*, a gene found upregulated in T-PLL which encodes a protein essential for thymocyte maturation and T-ALL pathogenesis [42]. Moreover, the expression of *SATB1* is induced upon hyperactivation of Wnt signaling in colorectal cancer [43], further supporting the study of this regulation also in T-PLL. Given the emerging role of circRNAs in cancer and, in particular, in hematological conditions in which circRNA detection was correlated with pathogenesis, chemotherapy resistance and prognosis [15,44,45], our findings could open new lines of investigation in T-PLL, aiming to clarify the contribution of aberrant circRNA expression to the maintenance of the malignant cell phenotype and the progression of disease.

A key point of this study is the exploration of the relationship between complex mutation patterns and gene and circRNA expression in T-PLL. The detection of variants from RNA-seq was able to identify both high- and low-VAF variants, and the robustness of the data was supported by Sanger validation of selected variants. However, RNA-seq-based variant calling is limited to expressed regions and may miss variants in low- or non-expressed genes; it can also be affected by transcript abundance, allele-specific expression, RNA-editing events, and splicing-related artifacts near intron–exon junctions. These issues were considered in our workflow through stringent filtering, including removal of known RNA-editing sites, exclusion of variants close to splice junctions, use of resources, and Sanger confirmation of selected variants. The detection of recurrent variants in the JAK–STAT axis (i.e., *STAT5B* and *JAK3* genes) further confirms its importance in T-PLL [8], which has been recently pharmacologically targeted with promising phase Ib results [46,47,48,49]. Along with the recurrent *ATM* and *KMT2C* mutations, these findings align with previously reported clustering of T-PLL mutated gene functions [7]. *ARID1A*, a novel gene recurrently mutated in our cohort, encodes a subunit of the chromatin remodeling complex and is mutated in several tumors for which clinically applicable drugs based on synthetic lethal strategy have been developed [50]. According to the recurrent oncogenic variants, a group of five genes (*STAT5B*, *JAK3*, *ATM*, *KMT2C*, and *ARID1A*) was thus examined to gain a better understanding of genotype/phenotype relations in T-PLL. To this end, we employed a linear model that linked each lesion to a set of genes or circRNAs showing significant expression changes between mutated and non-mutated samples. We considered only validated variants to ground on a solid basis, noting that *STAT5B*, *JAK3*, and *KMT2C* mutations were linked to similar expression patterns, while *ATM* mutations were associated with milder changes and *ARID1A* mutations to changes opposite to those seen in the other subgroups.

The strongest associations of our preliminary model of genotype-associated expression changes included the link between *STAT5B* lesions and the upregulation of lactotransferrin (LTF), an immune modulatory protein whose increased levels in T-ALL cell lines and in CLL inhibit ferroptosis [51]. *JAK3*-mutated cases displayed upregulation of *PLXNA4*, a semaphorin receptor, linked to immune modulation in melanoma [52], and downregulation of the chemokine receptor *CCR4*. *ATM* mutations were linked to the upregulation of the pro-proliferative interleukin-2 receptor subunit alpha (*IL2RA*), which is associated with aggressiveness in AML [53], and to the downregulation of *PYHIN1*, a cell cycle inhibitor [54]. *ARID1A* lesions were associated with the downregulation of *TLE4*, a transcription corepressor that negatively regulates the canonical Wnt signaling pathway and acts as a tumor suppressor in acute myeloid [55] and lymphoblastic leukemia [56]. We would like to highlight in patients with *KMT2C* mutations the upregulation of *TNFRSF4*, an oncogenic TNF-receptor, which is pharmacologically targeted in hematological cancers [57,58]. The sample size of this study prevents drawing definitive conclusions regarding genotype–phenotype relationships in T-PLL. Therefore, despite their compelling nature, our data about genotype–phenotype relations should be considered preliminary. Nevertheless, validation of the statistical model, together with the confirmation of the association between gene expression changes and oncogenic variants in the extended cohort, provides strong support for our findings.

## 5. Conclusions

In conclusion, we expanded the knowledge of gene expression aberrancies in T-PLL, presenting robust new data on lncRNAs and circRNAs with aberrant expression and their potential role in this malignancy. Moreover, we characterized the genetic makeup of T-PLL focusing on driver gene mutations and explored the association between gene and circRNA expression profiles and driver genes, offering valuable new clues into the disease’s heterogeneity, which may reflect new lines of investigation for the development of new therapeutic approaches. This approach could be effectively applied to larger cohorts in the future. Collaborative studies will be instrumental, especially for such a rare, incurable and enigmatic disease as T-PLL.

## Figures and Tables

**Table 1:** Clinical and molecular characteristics of the T-PLL cases profiled by RNA-seq. Clinical and molecular characteristics of the 10 T-PLL cases profiled by RNA-seq at diagnosis. Patients were subdivided by age, sex, treatment requirement, phenotypic analysis of the leukemic clone and TCR Vbeta expression. Not identified Vbeta means that the expansion was not recognizable, as it was not included within the approximately 70% of the TCR Vβ repertoire covered by the kit. Blood counts included white blood cells (WBC; 4.4–11 × 10^9^/L in CTR), hemoglobin (Hb; 140–175 g/L in CTR), absolute neutrophil count (ANC; 1.8–7.8 × 10^9^/L in CTR), lymphocyte (Ly; 1.1–4.8 × 10^9^/L in CTR) and platelets (PLT; 150–450 × 10^9^/L in CTR) counts. Mutational status of the hotspot regions of *STAT5B* and *JAK3* genes was determined by Sanger sequencing. NA: not available. More detailed features are reported in Supplementary Table S1.

Demographic	
Age (years)	69 mean; 46–86 range
Sex	5 female; 5 male
**Phenotype**	
CD4/7/25/26+ (number; %)	2/10 (20%)
CD4/7/25+ CD26− (number; %)	3/10 (30%)
CD4/7/26+ CD25− (number; %)	3/10 (30%)
CD4/7+ CD25/26− (number; %)	2/10 (20%)
**TCR Vbeta**	
Not identified	37.5%
Vbeta 1	12.5%
Vbeta 13.1	12.5%
Vbeta 5.3	12.5%
Vbeta 7.1	25%
**Blood counts at recruitment**	
WBC (×10^9^/L)	68.76 mean; 29.03–135.83 range
Hb (g/L)	121.7 mean; 81–150 range
ANC (×10^9^/L)	3.44 mean; 0.94–8.73 range; 3 NA
Ly (×10^9^/L)	51.58 mean; 25.58–84.31 range; 3 NA
PLT (×10^9^/L)	169.4 mean; 71–360 range
***STAT5B* screening**	
*STAT5B* mutated	6/10 (60%)
*STAT5B* wild-type	4/10 (40%)
***JAK3* screening**	
*JAK3* mutated	2/10 (20%)
*JAK3* wild-type	8/10 (80%)

## Data Availability

RNA-seq data (GSE281431) are freely available at Gene Expression Omnibus (https://www.ncbi.nlm.nih.gov/geo/, accessed on 26 July 2026).

## Supplementary Materials

The following supporting information can be downloaded at https://www.mdpi.com/article/10.3390/cancers18152442/s1. Supplementary Methods S1: Samples and mutation screening at diagnosis [59]; Supplementary Methods S2: Gene and circRNA expression estimation from RNA-seq [18,60,61]; Supplementary Methods S3: Oncogenic variant calling from RNA-seq [62,63,64,65,66,67,68,69,70,71,72,73]; Supplementary Methods S4: Functional enrichments [74,75,76,77]; Supplementary Methods S5: Modeling gene and circRNA expression relation with genetic lesions [78]; Figure S1: Gene loadings and KEGG pathway enriched in the principal component analysis (PCA) of T-PLL and control samples, according to gene expression profiles reported in the main Figure 1A; Figure S2: Validation of at least one representative variant per patient by Sanger sequencing; Figure S3: Gene and circRNA expression profile associated with distinct driver gene variants in T-PLL; Table S1: Clinical and molecular data of the T-PLL cohort; Table S2: Primers designed for RT-qPCR and Sanger sequencing; Table S3: Genes differentially expressed in T-PLL compared to CD4+ of healthy donors, with adjusted *p*-values (padj.) and Log Fold Changes (logFC); Table S4: CircRNAs differentially expressed in T-PLL compared to CD4+ of healthy donors, with adjusted *p*-values (padj.) and Log Fold Changes (logFC); Table S5: Expressed candidate driver variants detected in T-PLL patients and classified as oncogenic drivers according to Cancer Genome Interpreter (CGI) analysis; Table S6: List of the validated candidate variants detected in T-PLL patients; Table S7: Significant relations between genes hit by point mutations and phenotype, in terms of gene and circRNA expression profile.
