# Supplementary material for: Gene Expression, Non-Coding RNA, and Circular RNA Alterations in Patients with T-Prolymphocytic Leukemia
Source: Cancers (Basel). 2026 Jul 29;18(15):2442. doi: 10.3390/cancers18152442 (PMC13466056; doi:10.3390/cancers18152442)
Supplement: Supplementary file 1 [file cancers-18-02442-s001.zip › cancers-4426545-supplementary.pdf]

Gene expression, non-coding RNA, and circular RNA alterations in patients with T-Prolymphocytic Leukemia

Vanessa R. Gasparini [1,2,†], Silvia Orsi [1,3,†], Alessia Buratin [3,4], Elisa Rampazzo [1,2], Giulia Calabretto [1,2], Elena Buson [1,2], Alberto Caregari [3,4], Cristina Vicenzetto [5], Gregorio Barilà [6], Eleonora Roncaglia [3,4], Roberto Merlo [3,4], Livio Trentin [1], Monica Facco [1,2], Laura Pavan [6], Gianpietro Semenzato [2,*], Enrico Gaffo [3], Antonella Teramo [1,2], Renato Zambello [1,2,‡] and Stefania Bortoluzzi [7,*,‡]

1 Hematology Section, Department of Medicine, University of Padova, Padova, Italy, 35128.
2 Veneto Institute of Molecular Medicine (VIMM), Padova, Italy, 35129.
3 Department of Biology, University of Padova, Padova, Italy, 35131.
4 Department of Molecular Medicine, University of Padova, Padova, Italy, 35131.
5 Cardiology Section, Department of Cardiac Thoracic Vascular Sciences and Public Health, University of Padova, Padova, Italy, 35129.
6 Hematology Unit, San Bortolo Hospital, Vicenza, Italy, 36100.
7 Department of Surgery, Oncology and Gastroenterology, University of Padova, Padova, Italy, 35131.
† These authors contributed equally to this work.
‡ These authors also contributed equally to this work.
* Correspondence: stefania.bortoluzzi@unipd.it, Phone +39 049 827 650; g.semenzato@unipd.it, Phone +39 049 7923 236

SUPPLEMENTARY MATERIAL

Supplementary Methods

S1: Samples and mutation screening at diagnosis

Peripheral blood mononuclear cells (PBMC) of T-PLL patients were obtained after isolation with Ficoll-Hypaque (Sigma-Aldrich, Saint Louis, MO, USA) gradient centrifugation. PBMC were then stained with CD4, CD8, CD7, CD25 and CD26 antibodies and acquired with FACSCanto II (Becton Dickinson, Franklin Lakes, NJ, USA) to determine the immunophenotype of the leukemic clone. All samples presented high purity of the leukemic clone (>95% of lymphocytes and >95% of leukemic clone within the lymphocyte gate). Expression of cytotoxic markers (CD16, CD57 e CD56) was ruled out. To determine the presence of a clonal expansion, T-Cell Receptor (TCR) Gamma Gene Rearrangement Assay (Invivoscribe, San Diego, CA, USA) and IOTest Beta Mark Kit (Beckman Coulter, Brea, CA, USA) were performed. Sanger sequencing of hotspot *STAT5B* exons 16-18 was performed as previously described[1].

To collect the samples better representing the normal counterpart of T-PLL cells (CTR), PBMC from buffy coats of age-matched healthy donors were stained with the same panel of antibodies used for T-PLL samples. Then, healthy CD4+ cells were purified with magnetic Micro-Beads coated with monoclonal anti-human CD4 antibody (Miltenyi Biotec, Bergisch Gladbach, Germany) assessing purity (all samples >95%) by flow cytometry.

S2: Gene and circRNA expression estimation from RNA-seq

Expressed genes and circRNAs were detected with CircComPara2 v0.6.3[2], implementing data quality control and filtering, read alignment to the reference genome (GRCh38 v97) and linear and circular transcriptome reconstruction, respectively using Hisat2 and the combination of seven backsplicing detection methods. Genes with TPM $\geq$ 1 (Transcripts Per Million) and circRNAs detected by at least two methods in at least 10 samples or with at least 5 reads in at least 5 samples were kept. Differential expression analysis was performed using DESeq2 v.1.44.0[3] for genes and edgeR[4] v.4.2.0 for circRNAs and used a Benjamini-Hochberg adj. p-value $\leq$0.01 as threshold.

S3: Oncogenic variant calling from RNA-seq

Sequence reads were aligned to the human reference genome (hg38) by STAR v2.7[5] in Two-Pass mode. The alignments were optimized according to the gold standard procedure of Genome Analysis Toolkit v4.2.4 (GATK4) for variant calling from RNA-seq data[6]. Variant calling was performed with HaplotypeCaller[7] and Mutect2[6].

The variants were annotated with SnpEff[8] and SnpSift[9] using data available in dbSNP[10] (build 155), ClinVar[10,11], COSMIC[12] (v95), GnomAD[13] (v2.1.1), REDIportal[14] (hg38). Only variants supported by at least 5 reads, not occurring neither in previously annotated RNA editing sites nor at splice sites (within 5 nt, according to RegTools[15]), with a population allele frequency lower than 5%, and a variant allele frequency (VAF) in the tumor sample at least 5% and with predicted functional impact (HIGH or MODERATE) were kept. Finally, of these variants, only those classified as “driver” by Cancer Genome Interpreter[16] (CGI), were retained.

S4: Functional enrichments

Significantly enriched pathways (KEGG)[17] among differentially expressed genes or PC1 genes were identified using Gene Set Enrichment Analysis (GSEA)[18] with the gseKEGG function from the ClusterProfiler v.4.12.0[19] package.

For mutated genes, enriched pathways were determined through Overrepresentation Analysis (ORA)[20] and prioritized using the DICE similarity score via the BioCor package. The resulting dendrogram clustered mutated pathways based on similarity, further prioritizing them based on the variant's clonality and the biological significance of the mutated genes within these pathways.

S5: Modeling gene and circRNA expression relation with genetic lesions

To identify significant relations between genes hit by point mutations and phenotype, in terms of gene and circRNA expression profile, we fit a linear expression model using the Limma Bioconductor package[21].

For this analysis, we considered only highly expressed genes (Average TPM >4) and circRNAs (expressed with a read count of 8 in at least two samples). The expression matrices were merged and normalized using the Variance Stabilizing Transformation function from the DESeq2 package.

Considering the occurrence of mutations in 5 selected driver genes (*STAT5B, ATM, ARID1A, KMT2C*, and *JAK3*), the expression of gene/circRNA (GC) *k* in patient *i*, Y_{ik}, was modeled by the following equation:

$$Y_{ik} = \sum_{j=1} X_{ij}\beta_{jk} + \beta_{0k} + \epsilon \quad \text{(S1)}$$

where X_{ij} is the mutation matrix for patient *i* and mutation *j*, with entries X_{ij}=1 indicating that patient *i* has an oncogenic mutation *j* and 0 otherwise. The coefficients β_{jk} measure the expression change in gene/circRNA *k* linked to the presence of a mutation *j*. The entry β_{0k} denotes the baseline expression level of GC *k*. This model assumes that each mutation is associated with a certain set of GC expression changes and that the sample expression pattern is the sum of the changes linked to each mutation, when multiple are present.

Using this model, finding significant effects amounts to testing whether the coefficients β_{jk} are different from zero for each contrast (mutated vs non mutated samples). The Limma package uses a moderated t-statistic for these tests with a prior variance shared across genes and circRNAs. We used the Benjamini-Hochberg correction for multiple testing and an adj. p-value<0.05 as threshold. The accuracy of the applied tests and correction schemes was verified using a permutation approach in which each covariate was randomly permuted thereby breaking all correlations between genotype and expression.

Supplementary Tables

Supplementary Table S1. Clinical and molecular data of the T-PLL cohort. The table gives details regarding the clinical and molecular data at diagnosis of the 19 T-PLL patients enrolled for this study. Clinical data include immunophenotypic analysis of the leukemic clone, evaluation of the complete blood count data and treatment requirement. Molecular analysis comprises Sanger sequencing for evaluation of the hotspot region of *STAT5B* (exons 16-18) and *JAK3* (exons 11-15) genes.

→ see XLS file

Supplementary Table S2. Primers designed for RT-qPCR and Sanger sequencing. In this table are listed all the primers customized for the validations of mutational status by Sanger sequencing and differential expression of linear and circular transcripts by RT-qPCR. Fw: forward; Rv: reverse.

GENE	Target	Fw SEQUENCE (5'-3')	Rv SEQUENCE (5'-3')	METHOD
KAT6B	circRNA	CCTGTCAGCCTTCTACCCCA	GGAAAGACTGCATTCGGGTG	RT-qPCR
SATB1	circRNA	GCAACCAAAAGCCAGGTGAT	GGACCCTTCGGATCACTCAC	RT-qPCR
SEMA4B	circRNA	GCTTGCAAGGCGATGATGAC	CAGGAAGCTGAGGTTGCTACT	RT-qPCR
TGFBR3	circRNA	TGATTGCCATCTTTGCCCTG	GAGGGTCTGTGCTGCGTTA	RT-qPCR
TNRC6A	circRNA	CTTGTAACCACCATGCTGTGC	TTAGAGTTGGCAGGCTGAGG	RT-qPCR
ERBB3	mRNA	GCAGATCAGTGTGTAGCGTG	CGTGTGCAGTTGAAGTGACA	RT-qPCR
GAPDH	mRNA	AATGGAAATCCCATCACCATCT	CGCCCCACTTGATTTTGG	RT-qPCR
IL18	mRNA	GACGCATGCCCTCAATCC	CTAGAGCGCAATGGTGCAATC	RT-qPCR
IL1B	mRNA	TCCCCAGCCCTTTTGTTGA	TTAGAACCAAATGTGGCCGTG	RT-qPCR
NLRP3	mRNA	TGGAGTGTCGGAGAAGAG	TGCTGTCATTGTCCTGGTGT	RT-qPCR
PDGFB	mRNA	GACCCCAAACCCGAGGTT	ATGGTTGAGGAGGTGTTGACTT	RT-qPCR
LTF	mRNA	AGTCTACGGGACCGAAAGACA	CAGACCTTGCAGTTCGTTCAG	RT-qPCR
PLXNA4	mRNA	TGTGGAGTCCTGTGGTCAGT	CGGACACACTGCTTCATCTC	RT-qPCR
FIRRE	lncRNA	TGAAAGGGAATCCTGACGCC	TGCCTAGCTCTGACAATGGC	RT-qPCR
LINC00877	lncRNA	AGACTGGAAGTTTGGGGACAA	CCCCCTTGCATGTCCCATTA	RT-qPCR
LUCAT1	lncRNA	GCTCGGATTGCCTTAGACAG	GGGTGAGCTTCTTGTGAGGA	RT-qPCR
MIAT	lncRNA	GCTCACACCTCCTATTCCT	CTTCACCAACTCTCCCACT	RT-qPCR
TERC	lncRNA	GTGGTGGCCATTTTTTGTCTAAC	TGCTCTAGAATGAACGGTGGAA	RT-qPCR
NEAT1	lncRNA	GCGAGGTGCCTTTACTACAT	TGGAACCCAGAAGACAGA	RT-qPCR
ANK3_D609Y	Variant	TGCCTAAACTAGGAGGGTGC	CTGGTTGTTGTTTCTGCACG	Sanger
ANK3_R4369Q	Variant	TGAAAAGGAAAGGACGGGGA	TCCAGCCTCCAAGGATCAGA	Sanger
ARID1A_PQ1326-1327P	Variant	AGGGAAACATGAGCACTGGG	GTGGCGAAGCCTGATCCATA	Sanger
ATM_R3008C	Variant	ACTGTTCACCTCACTGAAACCT	ATCCTGGGAAAAGTCGGCTG	Sanger
ATM_S2394L	Variant	AGGCAGTAGAAGTTGCTGGAA	ACTGGTGAACATAAAATTGTCACT	Sanger
CMTR2_A171G	Variant	ACTCCCATCTGCAGTGACCA	CACCTTTGTGAAGCTCCAGG	Sanger
DNMT3A_R326C	Variant	GCATCCTAGCTCTCTGAGCC	CCTCGTGACCACTGTGTAATG	Sanger
JAK3_M511I	Variant	AACCCTTGGGGACTTTTCAC	TTTCTCTGCATCCACGACC	Sanger

JAK3_V674A	Variant	CTGTGTCTGGCCCCCTTAG	GGATCAGGGATCCACTTCCT	Sanger
KMT2C_R2463H	Variant	CGGCAGGAAATTAAAAGCATTACC T	AACCAGGCTTTCACCAGACC	Sanger
PTPRC_R682C	Variant	AACGGTCATTGGTAAGGGGG	CCAAGACAAACAAGGAGCTGAA	Sanger

Supplementary Table S3. Genes differentially expressed in T-PLL compared to CD4+ of healthy donors, with adjusted p-values (padj.) and Log Fold Changes (logFC).
→ see XLS file

Supplementary Table S4. CircRNAs differentially expressed in T-PLL compared to CD4+ of healthy donors, with adjusted p-values (padj.) and Log Fold Changes (logFC). For each group, the average expression at backsplice junction (BSJ) and the average circular to linear proportion (CLP) of each circRNA is also shown.
→ see XLS file

Supplementary Table S5. Expressed candidate driver variants detected in T-PLL patients and classified as oncogenic drivers according to Cancer Genome Interpreter (CGI) analysis.
→ see XLS file

Supplementary Table S6. List of the validated candidate variants detected in T-PLL patients. The 18 expressed putative somatic variants confirmed by Sanger sequencing in T-PLL patients subdivided by name of the gene, type of variant, position in the genome, genotype and patient who harbored the relative variant. All the variants resulted in heterozygosity except for *ANK3* R4369Q.

Gene	Variant	Position	Patient
ANK3	D609Y	chr10:60196207 C>A	T-PLL09
ANK3	R4369Q	chr10:60042719 C>T	T-PLL08
ARID1A	PQ1326-1327P	chr1:26773691-26773691 GCA>-	T-PLL01
ATM	R3008C	chr11:108365359 C>T	T-PLL06
ATM	S2394L	chr11:108329112 C>T	T-PLL03
CMTR2	A171G	chr16:71285409 G>C	T-PLL07
DNMT3A	R326C	chr2:25247629 G>A	T-PLL10
JAK3	M511I	chr19:17838299 C>T	T-PLL06
JAK3	M511I	chr19:17838299 C>T	T-PLL10
JAK3	V674A	chr19:17835109 A>G	T-PLL06
KMT2C	R2463H	chr7:152179888 C>T	T-PLL09
PTPRC	R682C	chr1:198732369 C>T	T-PLL06
STAT5B	N642H	chr17:42207711 T>G	T-PLL01
STAT5B	N642H	chr17:42207711 T>G	T-PLL02
STAT5B	N642H	chr17:42207711 T>G	T-PLL03
STAT5B	N642H	chr17:42207711 T>G	T-PLL05
STAT5B	N642H	chr17:42207711 T>G	T-PLL07
STAT5B	Y665F	chr17:42207641 T>A	T-PLL04

Supplementary Table S7. Significant relations between genes hit by point mutations and phenotype, in terms of gene and circRNA expression profile. Columns B-F show, for each gene/circRNA indicated in column A the coefficients obtained from the fitting of the linear regression for each mutated gene (columns B-F, refer respectively to STAT5B, JAK3, ARID1A, ATM, and KMT2C); the average expression in T-PLL, the R2 value associated (coefficient of determination; it indicates how well the linear regression model predicts gene/circRNA expression based on mutation status), plus the adjusted P-value for each pair of mutated genes and gene/circRNA expression (columns J-N) and the direction of significant relations (1 and -1, meaning the gene/circRNA tend to be upregulated and downregulated in samples with the driver gene mutation (columns O-S).

→ see XLS file

Supplementary Figures

Supplementary Fig. S1. Gene loadings and KEGG pathway enriched in the Principal Component analysis (PCA) of T-PLL and control samples according to gene expression profiles reported in the main Fig. 1A.

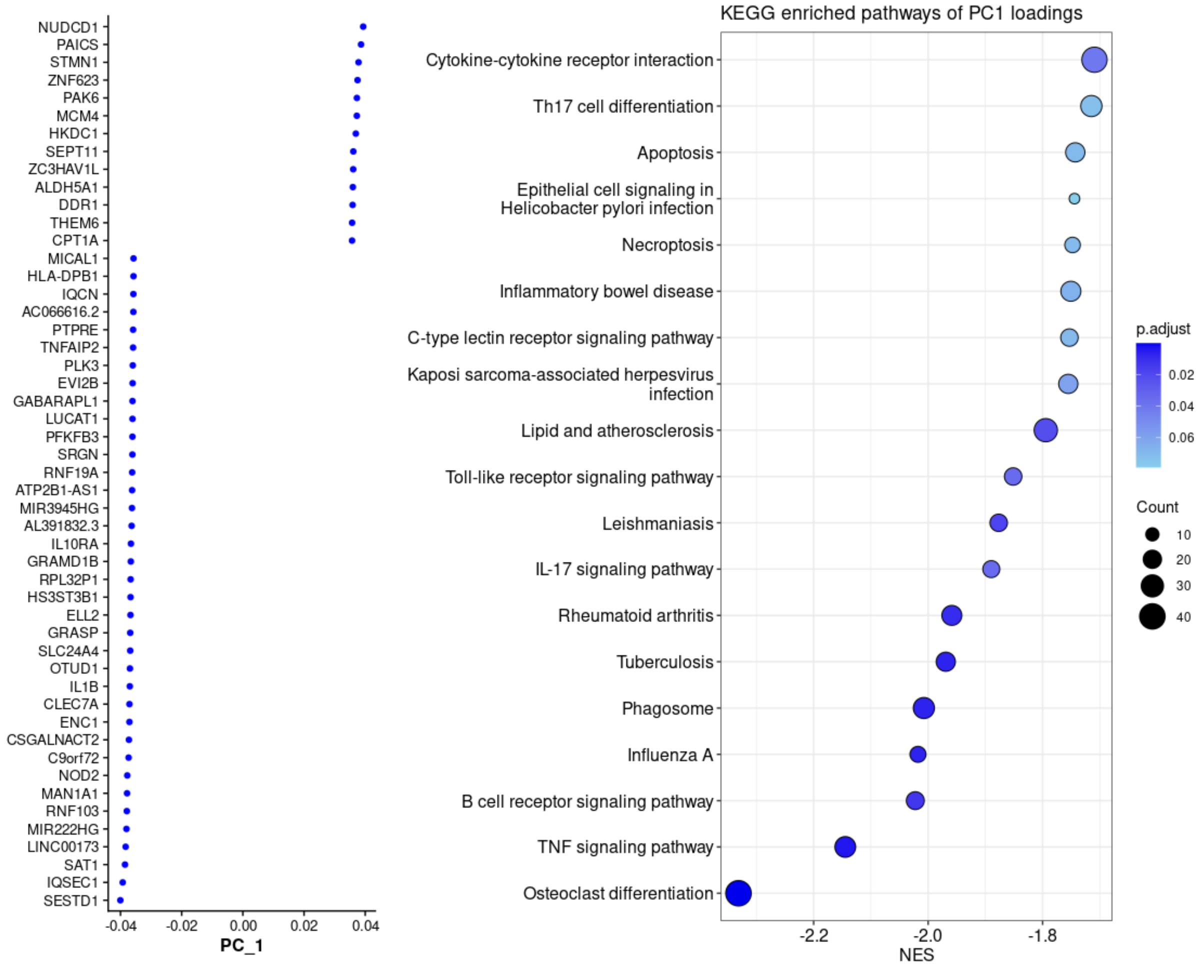

Supplementary Fig. S2. Validation of at least one representative variant per patient by Sanger sequencing. The blue arrow indicates the variant nucleotide; all mutations were found in heterozygosity except for T-PLL 08 which had the *ANK3* homozygous for the somatic variant.

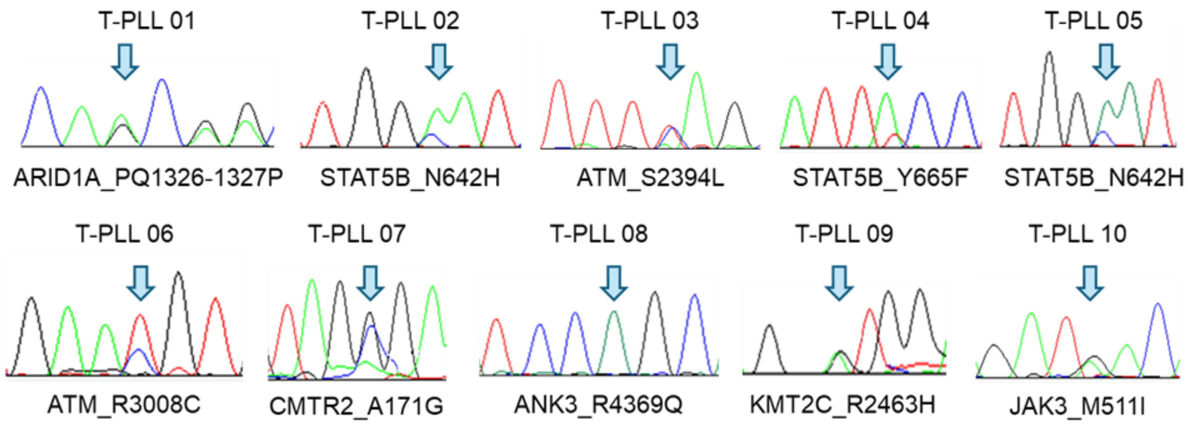

Supplementary Fig. S3. Gene and circRNA expression profile associated with distinct driver gene variants in T-PLL. Network representation of the strongest links detected between driver variants and gene and circRNA expression changes. For each mutated gene, the 50 most connected profiles according to the linear model are shown; edge colors indicate linear model β coefficients; genes and circRNAs highlighted in bold are significantly linked to only one mutated gene, according to the model.

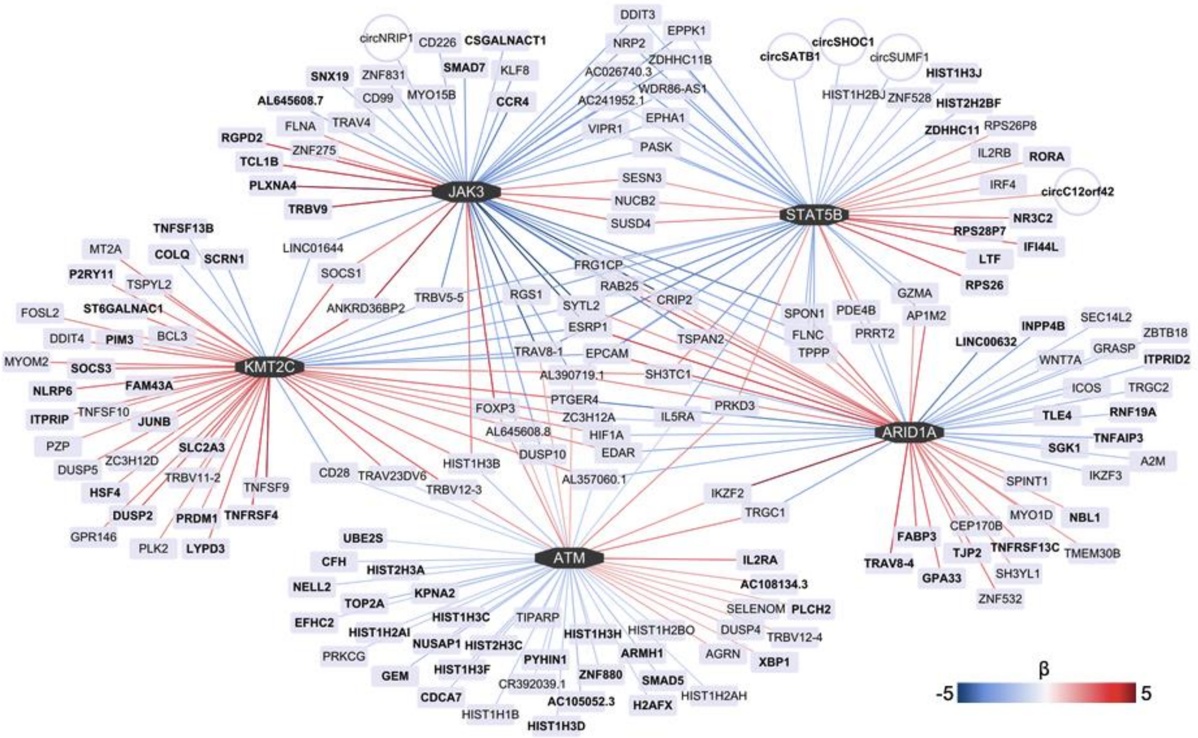

References

1 Gasparini VR, Binatti A, Coppe A, Teramo A, Vicenzetto C, Calabretto G *et al.* A high definition picture of somatic mutations in chronic lymphoproliferative disorder of natural killer cells. *Blood Cancer J* 2020; **10**: 42.

2 Gaffo E, Buratin A, Dal Molin A, Bortoluzzi S. Sensitive, reliable and robust circRNA detection from RNA-seq with CirComPara2. *Brief Bioinform* 2022; **23**. doi:10.1093/bib/bbab418.

3 Love MI, Huber W, Anders S. Moderated estimation of fold change and dispersion for RNA-seq data with DESeq2. *Genome Biol* 2014; **15**: 550.

4 Robinson MD, McCarthy DJ, Smyth GK. edgeR: a Bioconductor package for differential expression analysis of digital gene expression data. *Bioinformatics* 2010; **26**: 139–140.

5 Dobin A, Davis CA, Schlesinger F, Drenkow J, Zaleski C, Jha S *et al.* STAR: ultrafast universal RNA-seq aligner. *Bioinformatics* 2013; **29**: 15–21.

6 Van der Auwera GA, O'Connor BD. *Genomics in the Cloud: Using Docker, GATK, and WDL in Terra*. O'Reilly Media, 2020.

7 Poplin R, Ruano-Rubio V, DePristo MA, Fennell TJ, Carneiro MO, Van der Auwera GA *et al.* Scaling accurate genetic variant discovery to tens of thousands of samples. doi:10.1101/201178.

8 Cingolani P, Platts A, Wang LL, Coon M, Nguyen T, Wang L *et al.* A program for annotating and predicting the effects of single nucleotide polymorphisms, SnpEff: SNPs in the genome of Drosophila melanogaster strain w1118; iso-2; iso-3. *Fly* 2012; **6**: 80–92.

9 Cingolani P, Patel VM, Coon M, Nguyen T, Land SJ, Ruden DM *et al.* Using Drosophila melanogaster as a Model for Genotoxic Chemical Mutational Studies with a New Program, SnpSift. *Front Genet* 2012; **3**: 35.

10 Sherry ST. dbSNP: the NCBI database of genetic variation. Nucleic Acids Research. 2001; **29**: 308–311.

11 Landrum MJ, Lee JM, Benson M, Brown GR, Chao C, Chitipiralla S *et al.* ClinVar: improving access to variant interpretations and supporting evidence. *Nucleic Acids Res* 2018; **46**: D1062–D1067.

12 Tate JG, Bamford S, Jubb HC, Sondka Z, Beare DM, Bindal N *et al.* COSMIC: the Catalogue Of Somatic Mutations In Cancer. *Nucleic Acids Res* 2019; **47**: D941–D947.

13 Karczewski KJ, Francioli LC, Tiao G, Cummings BB, Alföldi J, Wang Q *et al.* The mutational constraint spectrum quantified from variation in 141,456 humans. *Nature* 2020; **581**: 434–443.

14 Picardi E, D'Erchia AM, Lo Giudice C, Pesole G. REDIportal: a comprehensive

database of A-to-I RNA editing events in humans. *Nucleic Acids Res* 2017; **45**: D750–D757.

15 Cotto KC, Feng Y-Y, Ramu A, Richters M, Freshour SL, Skidmore ZL *et al.* RegTools: Integrated analysis of genomic and transcriptomic data for the discovery of splice-associated variants in cancer. doi:10.1101/436634.

16 Tamborero D, Rubio-Perez C, Deu-Pons J, Schroeder MP, Vivancos A, Rovira A *et al.* Cancer Genome Interpreter annotates the biological and clinical relevance of tumor alterations. *Genome Med* 2018; **10**: 25.

17 Kanehisa M, Goto S. KEGG: kyoto encyclopedia of genes and genomes. *Nucleic Acids Res* 2000; **28**: 27–30.

18 Subramanian A, Tamayo P, Mootha VK, Mukherjee S, Ebert BL, Gillette MA *et al.* Gene set enrichment analysis: a knowledge-based approach for interpreting genome-wide expression profiles. *Proc Natl Acad Sci U S A* 2005; **102**: 15545–15550.

19 Wu T, Hu E, Xu S, Chen M, Guo P, Dai Z *et al.* clusterProfiler 4.0: A universal enrichment tool for interpreting omics data. *Innovation (Camb)* 2021; **2**: 100141.

20 Huang DW, Sherman BT, Lempicki RA. Bioinformatics enrichment tools: paths toward the comprehensive functional analysis of large gene lists. *Nucleic Acids Res* 2009; **37**: 1–13.

21 Smyth GK. Linear models and empirical bayes methods for assessing differential expression in microarray experiments. *Stat Appl Genet Mol Biol* 2004; **3**: Article3.
